# Effects of anti-malarial drugs on the electrocardiographic QT interval modelled in the isolated perfused guinea pig heart system

**DOI:** 10.1186/1475-2875-9-318

**Published:** 2010-11-10

**Authors:** Atsushi Kinoshita, Harumi Yamada, Hajime Kotaki, Mikio Kimura

**Affiliations:** 1 Division of Drug Informatics, Faculty of Pharmaceutical Sciences, Himeji Dokkyo University, 7-2-1 Kamiono, Himeji, Hyogo, 670-8524 Japan; 2 School of Pharmacy, International University of Health and Welfare, 2600-1 Kitakanemaru, Ootawara, Tochigi, 324-8501 Japan; 3 Shin-Yamanote Hospital, Japan Anti-Tuberculosis Association, 3-6-1 Suwa-cho, Higashi-Murayama, Tokyo, 189-0021 Japan

## Abstract

**Background:**

Concern over the potential cardiotoxicity of anti-malarial drugs inducing a prolonged electrocardiographic QT interval has resulted in the almost complete withdrawal from the market of one anti-malarial drug - halofantrine. The effects on the QT interval of four anti-malarial drugs were examined, using the guinea pig heart.

**Methods:**

The guinea pig heart was isolated, mounted on a Langendorff apparatus, and was then perfused with pyruvate-added Klebs-Henseleit solutions containing graded concentrations of the four agents such as quinidine (0.15 - 1.2 μM), quinine (0.3 - 2.4 μM), halofantrine (0.1 - 2.0 μM) and mefloquine (0.1 - 2.0 μM). The heart rate-corrected QaTc intervals were measured to evaluate drug-induced QT prolongation effects.

**Results:**

Quinidine, quinine, and halofantrine prolonged the QaTc interval in a dose-dependent manner, whereas no such effect was found with mefloquine. The EC_50 _values for the QaTc prolongation effects, the concentration that gives a half-maximum effect, were quinidine < quinine ≈ halofantrine.

**Conclusions:**

In this study, an isolated, perfused guinea pig heart system was constructed to assess the cardiotoxic potential of anti-malarial drugs. This isolated perfused guinea pig heart system could be used to test newly developed anti-malarial drugs for their inherent QT lengthening potential. More information is required on the potential variation in unbound drug concentrations in humans, and their role in cardiotoxicity.

## Background

Worldwide, in 2006, an estimated 247 million (189-327 million) malaria cases occurred, with an approximated 881,000 (610,000-1,212,000) deaths [1]. *Plasmodium falciparum *is the species that can cause severe, complicated malaria and death. Intravenous (IV) quinine (a 4-quinoline methanol) has been the mainstay of treatment for such severe malaria, although in some countries, including the United States, quinidine, the dextrorotatory diastereoisomer of quinine, is used because of the non-availability of IV quinine. Parenteral forms of artemisinin derivatives are increasingly being used in developing countries and more recently also in industrialized countries. Cardiac toxicity has been a major concern with the use of IV quinine or quinidine, with quinidine considered to be more toxic than quinine [2,3]. The primary mechanism of cardiotoxicity caused by quinine or quinidine is the prolongation of the electrocardiographic (ECG) QT interval which can cause potentially fatal ventricular arrhythmias, including torsades de pointes, and even sudden death.

Since the 1960s, chloroquine-resistant and multidrug-resistant strains of *P. falciparum *have emerged in Africa and Southeast Asia and have spread worldwide. Newer anti-malarial drugs were developed including mefloquine, a 4-quinoline methanol similar to quinine, and halofantrine, a 9-phenanthrene methanol structurally related to quinoline anti-malarial drugs. Because both drugs are administered orally, their widespread use was anticipated for the treatment of uncomplicated cases of drug-resistant *P. falciparum *infection. However, in 1993, reports of severe and sometimes fatal cardiotoxicity associated with the use of halofantrine led the World Health Organization to limit its use [4], and as of 2002, there were at least 20 reports of fatal cardiac complications relating to use of the drug [5]. These events were attributed to a QT prolongation effect of halofantrine, identified in several human studies of the drug [6,7]. These unexpected cardiac problems resulted in the withdrawal of the drug from the market in many countries except Pakistan and parts of West and Central Africa [8], and underlines the importance of examining the cardiotoxic potential of quinoline and other structurally related anti-malarial drugs before the wider marketing of newer drugs.

In this study, the effects on the QT interval of the following anti-malarials: quinidine, quinine, halofantrine, and mefloquine were examined, using an isolated perfused guinea pig heart model. The aim of this study was to clarify whether the results obtained from this model could be used to predict the cardiotoxicity of these anti-malarial drugs when used in clinical settings.

## Methods

### Chemical agents

Quinidine sulfate dihydrate and quinine hydrochloride dihydrate were purchased from Wako Pure Chemical Industries Ltd. (Tokyo, Japan). Mefloquine hydrochloride and halofantrine hydrochloride were kindly donated by Roche Co. Ltd. (Basel, Switzerland) and SmithKlein Beecham Co. Ltd. (Brentford, UK), respectively. All other chemicals used were of the reagent grade and were purchased commercially.

### Isolation of guinea pig hearts

The isolated perfused guinea pig heart system used in this study was constructed using a method described elsewhere [9]. Male Hartley guinea pigs weighing 350 to 500 g were anesthetized with a mixture of urethane and α-chloralose (1.2 and 30 mg/kg, respectively, intraperitoneally), and then injected with heparin (500 U/body, intraperitoneally). After 30 min, the heart was promptly excised. After the aorta was cannulated, the heart was mounted on a Langendorff apparatus and perfused with the pyruvate-added Klebs-Henseleit solution composed of: NaCl, 118 mM; KCl, 4.7 mM; CaCl_2_, 2.55 mM; MgSO_4_, 1.18 mM; KH_2_PO_4_, 1.18 mM; NaHCO_3_, 24.88 mM; glucose, 11.1 mM; sodium pyruvate, 2 mM; ascorbic acid, 0.14 mM; EDTA2Na, 0.5 mM. The solution was aerated with O_2_: CO_2 _(95:5) and kept at 37°C (pH 7.4 ± 0.01). The perfusion pressure was kept at 85 cm of water. The sinoatrial node of the heart was crushed after perfusion was commenced.

### Electrocardiogram recording

An ECG recording of the epicardial surface was commenced immediately after attaching the heart to the Langendorff apparatus. A stimulator was seated at the right atrium and the heartbeats were artificially kept constant at 210 per min by 3.5 Hz stimuli. Two silver wire electrodes were placed on the epicardial surface. Signals from both electrodes were amplified by an electric amplifier (AB-621G, Nihon-Kohden, Tokyo), recorded on a personal computer (PC-9801VX, NEC, Tokyo) *via *an A/D converter (Analog-Pro Jr., Canopus Electric, Kobe), and analysed with WAVE MASTER II and WM Read (Canopus Electric, Kobe) as described previously [10,11].

### QT interval measurement

Quinidine, quinine, and mefloquine were dissolved in Klebs-Henseleit solution at 0.15 - 1.2 μM, 0.3 - 2.4 μM, 0.1 - 2.0 μM, respectively. Halofantrine, which is poorly water-soluble, was first dissolved in polyethylene glycol 400 (PEG) and then in Klebs-Henseleit solution at 0.1 - 2.0 μM, with a final PEG concentration of 0.1% (v/v). The anti-malarial drug free 0.1% PEG Klebs-Henseleit solution served as a control for halofantrine treatment. Each heart was allowed to equilibrate with the drug-free solutions for 30 min. Measurements were performed after perfusion with the drug-containing solutions for 15 min. ECG parameters such as the heart rate, QT or QaT (from the beginning of the Q wave to the top of the T wave) intervals were obtained from the average wave shape of recordings for 10 sec.

### Analysis of the QT interval prolongation

QTc and QaTc intervals were obtained after correction of QT and QaT intervals using the Bazett's formula [12], since the formula was shown to be applicable to the guinea pig heart [13]. The QT prolongation effects of each drug were fitted simultaneously according to the full nonlinear regression analysis for effect as expressed in the equation below.

$$E=\frac{E_{\max}}{1+exp^{{}^{-}(\frac{C-EC_{50}}{E_{\min}})}}$$

Where E is the change in QaTc interval, E_max _is the maximum effect of the drug, E_min _is the minimum effect of the drug, EC_50 _is the concentration that gives a half-maximum effect, and C is the concentration of drug. Each parameter was calculated by the simultaneous fitting using a nonlinear least-squares programme (MULTI) with the modified Marquardt method [14].

### Statistics

Change in the QaTc interval is expressed as a mean ± standard error of the mean (SEM) and EC_50 _is expressed as a mean ± standard deviation (SD). Statistical analysis was performed using the Student's *t*-test.

## Results

### Perfusion with quinidine

Perfusion of the isolated guinea pig heart with the control solutions showed no QTc prolongation effect. To assess the optimal experimental conditions, the hearts were perfused with graded concentrations of quinidine (the prototype anti-malarial drug carrying cardiotoxic potential). Quinidine prolonged both the QTc and QaTc intervals at concentrations of 0.15 - 1.2 μM in a dose dependent manner. The EC_50 _for the QTc and the QaTc interval was 0.45 and 0.49 μM, respectively. Unexpectedly, the QTc interval proved difficult to measure at high drug concentrations as the end of the T-wave was unclear, overlapping the following P-wave. Therefore, it was decided to use the QaTc instead of the QTc interval to assess drug-induced QT lengthening throughout this study.

### Effects of anti-malarial drugs on the QaTc interval

**Figure 1:**
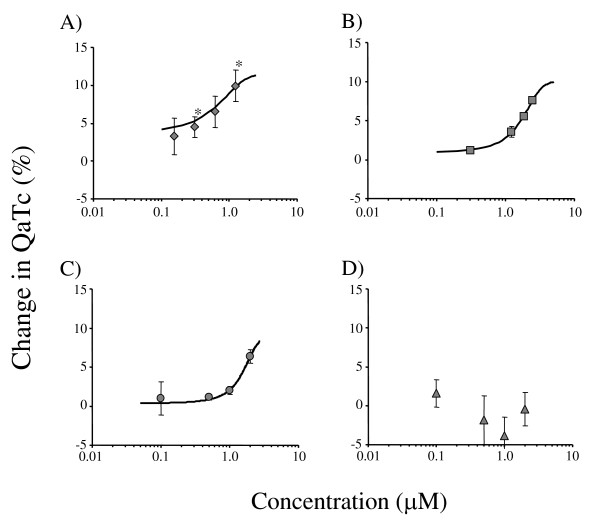
**Relationships between QaTc changes and concentrations of quinidine (A), quinine (B), halofantrine (C), and mefloquine (D) in the isolated perfused guinea pig heart system**. Each bar represents the mean ± SEM (n = 3 - 4). *Statistically significant compared with the same concentrations of quinine (p < 0.05).

Figure 1 shows the relationship between concentrations of the four anti-malarial drugs and the changes in the QaTc interval. Quinidine and quinine prolonged the QaTc interval in a dose dependent manner within the range of concentrations described above. Statistically, the QaTc prolongation effect was significantly higher with quinidine than quinine at concentrations of 0.3 and 1.2 μM (*p *<0.05). Halofantrine also prolonged the QaTc interval in a dose dependent manner within the range of concentrations described above. The EC_50 _values of quinidine, quinine, and halofantrine were 0.49 ± 0.61, 1.68 ± 0.43, 1.59 ± 1.26 μM, respectively, and thus the *in vitro *QaTc prolongation effect was highest with quinidine, and those with quinine and halofantrine were similar and were lower than that with quinidine. In contrast, mefloquine did not prolong the QaTc interval within the range of concentrations of 0.5 - 2.0 μM.

## Discussion

An isolated, perfused guinea pig heart system was constructed to assess the cardiotoxic potential of anti-malarial drugs. While the cardiotoxic effects of halofantrine have been studied previously using a similar (feline heart) model [15], there have been no previous studies reported where the cardiotoxic potential of several anti-malarial drugs were assessed simultaneously.

Quinidine, quinine, and halofantrine, all of which have been known to have potential cardiotoxicity in humans, exerted a QT prolongation effect in this experimental model. The effects of the three anti-malarial drugs were shown to be dose-dependent at the drug concentrations tested. In general, the biologically active component is considered to be the unbound drug, and the therapeutic range of unbound quinine in African children was shown to be 0.2 - 2.0 mg/mL (0.62 - 6.2 μM) [16]. This suggests that the quinine concentrations used in this experimental model may prove clinically relevant. The observed higher QT prolongation effect of quinidine (EC_50 _= 0.49 ± 0.61 μM) over quinine (1.68 ± 0.43 μM) is consistent with the findings in humans that the former is more cardiotoxic than the latter in terms of developing serious ventricular arrhythmias [2] and prolonging the QT interval [3]. An *in vitro *study was conducted on the inhibition of potassium channel currents on *Xenopus *oocytes expressing the human *ether-a-go-go*-related gene (hERG), which represents an underlying molecular mechanism of QT prolongation [17]. This study also showed that quinidine was more toxic than quinine with IC_50 _values of 4.6 μM and 57 μM, respectively. In addition to this inherent difference in cardiotoxic potential between quinidine and quinine, the former was reported to have a higher unbound fraction than the latter [18]. When assessing cardiotoxicity in human therapies, the possibility of finding differences in unbound fractions of an anti-malarial drug should also be considered. For example, the plasma unbound fraction of quinine was reported to be lower in cerebral malaria than in uncomplicated malaria [19], which may explain why severe quinine toxicity is unusual in severe falciparum malaria [20]. Moreover, studies are needed to investigate the various factors that could influence the total concentration and consequently the unbound concentration of an anti-malarial drug to ensure safer use of drugs.

Mefloquine did not prolong the QT interval within the range of concentrations of 0.5 - 2.0 μM. This agent is characterized by its very high protein binding, e.g, 98.3% shown in humans [21], with the unbound plasma mefloquine concentration in human therapy reported to be 0.05 μM [22]. This concentration is below the lowest concentration used in this study, making it very unlikely to be cardiotoxic in clinical settings. In fact, although Davis *et al *[23] suggested a mild and transient QTc lengthening in humans after mefloquine use, this was not evident in other studies [6,24], consistent with the expert's view that there is no convincing evidence for significant cardiotoxicity following mefloquine administration [2]. The possible mefloquine-induced cardiotoxicity might, however, be due to another mechanism, i.e., reduced contractility of cardiac muscles due to inhibition of the L type Ca^2+ ^channel by mefloquine [25].

The findings of this study, which showed that the QT prolongation effect of halofantrine was lower than that of quinidine and similar to that of quinine, may not seem to be clinically relevant. There are, however, few studies in which the therapeutic levels of unbound halofantrine concentrations in humans are well defined. One study showed the average peak total halofantrine concentration to be 6.4 μM [26], and the serum protein binding rate of halofantrine was reported to be 83% [27]. Therefore, the average peak unbound halofantrine concentration was calculated as 1.2 μM.

Other reports showed that the unbound therapeutic plasma concentration was 0.57 μM [22]. According to the results of this study, these reportedly low concentrations in humans do not seem to lengthen the QT interval. However, halofantrine is characterized by its marked differences in plasma concentrations among individuals, with one individual showing a five-times higher peak concentration than the other [26]. It has also been reported that its absorption is significantly enhanced when administered with fatty food (6.6 times higher peak concentrations) [28] or grapefruit juice [29]. In addition, the metabolite desbutylhalofantrine was shown to have some QT interval prolongation effect in a rabbit model [30]. Alternatively, the inherent cardiotoxicity of halofantrine may be detected more sensitively in other experimental models. For example, another *in vitro *study of inhibition of the potassium channel currents on hERG transfected cells, the IC_50 _was as low as 0.04 μM for halofantrine whereas it was 2.6 μM for mefloquine [22].

To assess cardiotoxicity of an anti-malarial drug, factors other than the inherent QT prolongation potential as shown in this study and the plasma unbound drug concentration need to be considered. For instance, accumulation in the myocardium may differ between anti-malarial drugs [22]. Furthermore, one report showed an absence of QT prolongation with an increased fraction of unbound quinidine induced by heparin administration [31]. Therefore, caution must be exercised when simply applying findings of unbound drug levels to assess cardiotoxicity in clinical settings.

## Conclusion

The results of this study were largely consistent with the reported cardiotoxicity of the four anti-malarial drugs in clinical use. This isolated perfused guinea pig heart system could be used to test newly developed anti-malarial drugs for their inherent QT lengthening potential. More knowledge is required on the variability of unbound anti-malarial drug concentrations in humans, as well as their impact on cardiotoxicity in clinical settings.

## Competing interests

The authors declare that they have no competing interests.

## Authors' contributions

AK and HY contributed to the study design, participated in the acquisition and interpretation of data, performed the statistical analysis, and helped draft the manuscript. HK and MK contributed to the acquisition and interpretation of data, and helped review the manuscript. All authors read and approved the final manuscript.
